# The Flavonoid Apigenin Ameliorates Cisplatin-Induced Nephrotoxicity through Reduction of p53 Activation and Promotion of PI3K/Akt Pathway in Human Renal Proximal Tubular Epithelial Cells

**DOI:** 10.1155/2015/186436

**Published:** 2015-05-21

**Authors:** Sung Min Ju, Jun Gue Kang, Jun Sang Bae, Hyun Ock Pae, Yeoung Su Lyu, Byung Hun Jeon

**Affiliations:** ^1^Department of Pathology, College of Korean Medicine, Wonkwang University, Iksan, Jeonbuk 570-749, Republic of Korea; ^2^Department of Pathology, Chonbuk National University Medical School, Research Institute of Clinical Medicine and Institute for Medical Sciences, Jeonju, Jeonbuk 561-756, Republic of Korea; ^3^Department of Microbiology and Immunology, Wonkwang University School of Medicine, Iksan, Jeonbuk 570-749, Republic of Korea; ^4^Department of Neuropsychiatry, College of Korean Medicine, Wonkwang University, Iksan, Jeonbuk 570-749, Republic of Korea; ^5^Research Center of Traditional Korean Medicine, Wonkwang University, Iksan, Jeonbuk 570-749, Republic of Korea

## Abstract

Apigenin is a member of the flavone subclass of flavonoids present in fruits and vegetables. Apigenin has long been considered to have various biological activities, such as antioxidant, anti-inflammatory, and antitumorigenic properties, in various cell types. Cisplatin was known to exhibit cytotoxic effect to renal cells by inducing apoptosis through activation of p53. The present study investigated the antiapoptotic effects of apigenin on the cisplatin-treated human renal proximal tubular epithelial (HK-2) cells. HK-2 cells were pretreated with apigenin (5, 10, 20 *μ*M) for 1 h and then treated with 40 *μ*M cisplatin for various times. Apigenin inhibited the cisplatin-induced apoptosis of HK-2 cells. Interestingly, apigenin itself exerted cytostatic activity because of its ability to induce cell cycle arrest. Apigenin inhibited caspase-3 activity and PARP cleavage in cisplatin-treated cells. Apigenin reduced cisplatin-induced phosphorylation and expression of p53, with no significant influence on production of ROS that is known to induce p53 activation. Furthermore, apigenin promoted cisplatin-induced Akt phosphorylation, suggesting that enhanced Akt activation may be involved in cytoprotection. Taken together, these results suggest that apigenin ameliorates cisplatin-induced apoptosis through reduction of p53 activation and promotion of PI3K/Akt pathway in HK-2 cells.

## 1. Introduction

Cisplatin, which is also named* cis*-diammineplatinum (II) dichloride, is called the “penicillin of cancer” because it is a widely used chemotherapeutic agent employed for treatment of human cancers [1, 2]. However, the chemotherapeutic use of cisplatin is limited by severed side effects, such as nephrotoxicity, neurotoxicity, ototoxicity, and emetogenicity [3–6]. Among them, nephrotoxicity has been reported as the major limiting factor in cisplatin chemotherapy of cancer patients [1, 2]. Because of the importance of cisplatin chemotherapy in cancer patients, many studies have focused on protective strategies to alleviate cisplatin nephrotoxicity [7]. Reactive oxygen species (ROS) and p53 tumor suppressor protein are leading causes of cisplatin nephrotoxicity [8]. Previous reported studies have shown that cisplatin induces apoptotic cell death in renal cells through mechanism of ROS-mediated p53 activation [9, 10]. NAC (*N*-acetyl-cysteine), a scavenger of ROS, and pifithrin-*α*, a pharmacological inhibitor of p53, inhibit cisplatin-induced apoptosis by reducing the activation of p53 in renal cells, and this suggests that inhibiting the p53 activation may be an important target to alleviate cisplatin nephrotoxicity.

Apigenin (4′,5,7-trihydroxylflavone), found in fruits and vegetables, such as onions, oranges, parsley, and chamomile, is a member of the flavone subclass of flavonoids that are the aglycone of several important glycosides [11–13]. Apigenin has various biological activities, including antioxidant, anti-inflammatory, antimutagenic, and anti-tumorigenic properties [14–17]. Particularly, anticancer activity of apigenin has been widely reported in a wide variety of tumor types [17–21]. Apigenin has low toxicity and has been shown to exert selective effects in inhibiting cell growth and inducing apoptosis in cancer cells without affecting normal cells [21]. Also, recent studies show that apigenin enhances the chemosensitivity to cisplatin in carcinoma cells [22, 23]. However, the effects of apigenin on cisplatin nephrotoxicity have not been investigated. In the present study, we demonstrated that apigenin is capable of ameliorating cisplatin-induced nephrotoxicity in human renal proximal tubular epithelial (HK-2) cells.

## 2. Materials and Methods

### 2.1. Reagent and Antibodies

4′,5,7-Trihydroxyflavone (Apigenin; Cat. number A3145; ≥97% purity),* cis*-diammineplatinum(II) dichloride (Cisplatin), 2′,7′-dichlorofluorescin diacetate (DCFH-DA), dimethyl sulfoxide (DMSO), 3-(4,5-dimethylthiazol-2-yl)-2,5-diphenyl tetrazolium bromide (MTT), propidium iodide (PI), and ribonuclease A (RNase A) were purchased from Sigma-Aldrich (St. Louis, MO, USA). Wortmannin was purchased from Calbiochem (La Jolla, CA, USA). Dulbecco's modified Eagle's medium (DMEM)/F12, trypsin-EDTA, fetal bovine serum (FBS), and antibiotic-antimycotic solution were purchased from GIBCO (Grand Island, NY, USA). RIPA lysis buffer and halt protease and phosphatase inhibitor cocktail were purchased from Thermo Fisher Scientific, Inc. (Waltham, MA, USA). Colorimetric caspase-3 assay kit was purchased from Abcam (Cambridge, MA, USA). Anti-cleaved caspase-3 (p20), anti-PARP, anti-Akt, anti-phospho-Akt (Ser473), anti-p53, and anti-phospho-p53 (Ser15) antibodies were purchased from Cell Signaling Technology (Beverly, MA, USA). Anti-*β*-actin antibody was purchased from Santa Cruz Biotechnology (Santa Cruz, CA, USA). HRP-conjugated goat anti-rabbit IgG and rabbit anti-mouse IgG antibodies were purchased from Invitrogen (Burlington, ON, Canada).

### 2.2. Cell Culture and Treatment

Immortalized human renal proximal tubular epithelial (HK-2) cells were obtained from the American Type Culture Collection (ATCC, Manassas, VA, USA). The cells were maintained in DMEM/F12 (1 : 1) supplemented with 10% FBS in 1 : 100 dilution of an antibiotic-antimycotic solution at 37°C in a 5% CO_2_ incubator. Exponentially growing cells were seeded into a culture dish at 1 × 10^5^ cells/mL in complete medium for 24 h before treatment with chemicals. The cells were pretreated with apigenin (5, 10, 20 *μ*M) for 1 h and then treated with or without 40 *μ*M cisplatin for indicated times under serum-free condition.

### 2.3. Cell Viability Assay

Cell viability was determined by the colorimetric assay using 3-(4,5-dimethylthiazol-2-yl)-2,5-diphenyl tetrazolium bromide (MTT). HK-2 cells were seeded into 12-well plates at 1 × 10^5^ cells/well. After treatment, the cells were washed with serum-free medium, and 1 mL of MTT solution (0.5 mg/mL in serum-free medium) was added to each well. After incubation for 4 h at 37°C, MTT containing medium was then removed by aspiration. The blue formazan product generated was dissolved by the addition of 500 *μ*L of 100% dimethyl sulfoxide (DMSO) per well. The amount of formazan was determined at 570 nm using SpectraMAX 250 microplate reader (Molecular Devices, Sunnyvale, CA, USA). The percent of cell proliferation was calculated using the following equation: (mean OD of treated cells/mean OD of control cells) × 100.

### 2.4. Analysis of Cell Morphology and Cell Cycle

HK-2 cells were pretreated with 20 *μ*M apigenin for 1 h and treated with or without 40 *μ*M cisplatin for 24 h. Cellular morphological changes were observed under inverted microscope (DIAPHOT 300, Nikon, Japan). Cell cycle progression was monitored by quantitating cellular DNA content after staining with the DNA-binding dye propidium iodide (PI). Cells were harvested, washed with PBS (pH 7.4), and fixed with ice-cold 70% ethanol at 4°C for 1 h. After removing the ethanol by repeated washing in PBS (pH 7.4), Cells were resuspended in 1 mL of PI (10 *μ*g/mL) solution containing 100 *μ*g/mL RNase A, incubated at 37°C for 1 h in the dark condition, and then analyzed on a FACS Calibur. Cell cycle analysis determined DNA content from fixed cells stained with PI.

### 2.5. Caspase-3 Activity Assay

The activity of caspase-3 was determined by colorimetric caspase-3 assay kit according to the manufacturer's protocol. Briefly, cells were harvested after treatment, washed with PBS (pH 7.4), and then lysed in lysis buffer. Cell lysates were centrifuged at 10,000 ×g for 1 min, and 50 *μ*L of extracts containing 50 *μ*g protein was incubated with 50 *μ*L of 2x reaction buffer and 5 *μ*L of 4 mM DEVD-*p*-NA substrate at 37°C for 2 h in the dark condition. The colorimetric release of* p*-nitroaniline from DEVD-*p*-NA substrate was measured at 405 nm using SpectraMAX 250 microplate reader.

### 2.6. Reactive Oxygen Species Production Assay

The relative levels of reactive oxygen species (ROS) were determined by 2′,7′-dichlorofluorescin diacetate (DCFH-DA) fluorescence assay. HK-2 cells were seeded into 96-well microplates (1 × 10^4^ cells/well) for 24 h and incubated in HBSS (pH 7.4) solution containing 20 *μ*M DCFH-DA at 37°C for 1 h. After incubation, cells were washed by HBSS (pH 7.4) and treated with 40 *μ*M cisplatin under HBSS (pH 7.4) for the indicated time periods. Relative DCF fluorescence intensity was determined with excitation wavelength of 485 nm and emission wavelength of 524 nm using SpectraMAX M2 multimode microplate reader (Molecular Devices, Sunnyvale, CA, USA).

### 2.7. Western Blot Analysis

The cells were lysed in RIPA lysis buffer containing 1% halt protease and phosphatase inhibitor cocktail. Cell lysates were centrifuged at 20,000 ×g for 15 min at 4°C, and the protein concentration was determined using a Bradford assay. Samples containing 50 *μ*g of total protein were resolved by SDS-PAGE gel and transferred onto a nitrocellulose membrane for 3 h at 40 V. The membranes were blocked with Tris-buffered saline with Tween-20 (20 mM Tris-HCl, pH 7.6, 150 mM NaCl, 0.05% Tween-20) containing 5% nonfat dry milk and probed with primary antibodies (all 1 : 1000 in 3% BSA in Tris-buffered saline with 0.05% Tween-20) overnight at 4°C with gentle shaking. Protein spots were detected using HRP-conjugated secondary antibodies (all 1 : 2000 in 3% BSA in Tris-buffered saline with 0.05% Tween-20). Immunoreactive bands were visualized using the SuperSignal West Pico Chemiluminescent Substrate Kit (Thermo Fisher Scientific, Inc. Waltham, MA, USA) and then developed using the FluorChem E System (ProteinSimple, San Jose, CA, USA). The density of bands was quantitated using Quantity One 4.6.6 software (Bio-Rad, Hercules, CA, USA).

### 2.8. Statistical Analysis

Statistical analysis was performed using Microsoft Office Excel 2010 (Microsoft, Redmond, WA). The data were expressed as means ± standard deviation (SD). The statistically significant differences between two groups were calculated by Student's *t*-test. *p* value <0.05 was considered to indicate statistically-significant differences.

## 3. Results

### 3.1. Apigenin Reduces Cisplatin-Induced Cytotoxicity in HK-2 Cells

**Figure 1:**
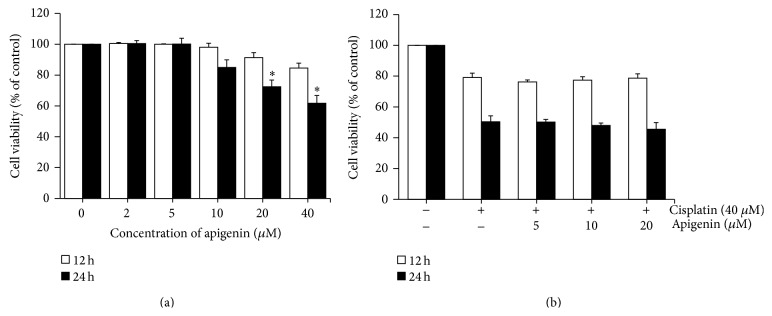
The effects of apigenin on cell viability in cisplatin-treated HK-2 cells. Cells were incubated for 12 and 24 h with different concentrations of apigenin (a). Cells were pretreated with different concentrations of apigenin for 1 h and then exposed to 40 *μ*M of cisplatin for 12 and 24 h (b). Cell viability was measured by MTT assay. Values are means ± SD, *N* = 3. ^*∗*^ *p* < 0.05 versus untreated cells.

After incubation with different concentrations of apigenin for indicated times, the viability of HK-2 cells was determined using the MTT assay. There was a decrease in cell viability following apigenin exposure (Figure 1(a)). After incubation with 20 and 40 *μ*M of apigenin for 24 h, cell viability was significantly reduced to approximately 72% and 61% of control levels, respectively. To evaluate the effects of apigenin on cell viability of cisplatin-treated HK-2 cells, the cells were incubated with 40 *μ*M of cisplatin for 12 and 24 h after pretreatment with different concentrations of apigenin (5–20 *μ*M). Results showed that apigenin had no obvious effect on cell viability of cisplatin-treated cells at 12 and 24 h (Figure 1(b)).

**Figure 2:**
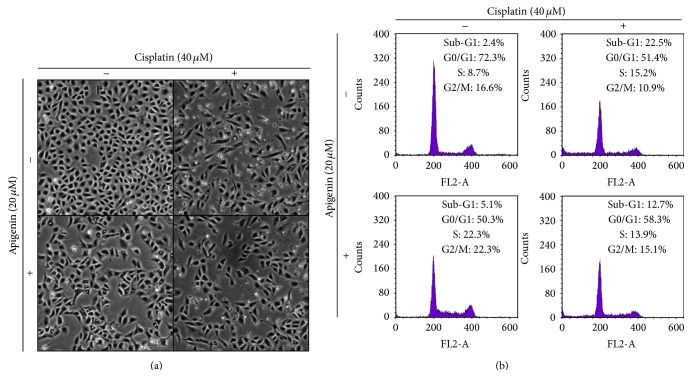
The effects of apigenin on cell morphology and cell cycle progression in cisplatin-treated HK-2 cells. Cells were treated with 40 *μ*M of cisplatin for 24 h in the absence or presence of 20 *μ*M of apigenin (pretreatment for 1 h). Cell morphology was observed using an inverted microscope (a). Cell cycle progression was analyzed by a flow cytometry (b).

We next examined whether the apigenin might have the cytoprotective effect on cisplatin-induced cytotoxicity in HK-2 cells. The cells were treated with 40 *μ*M of cisplatin for 24 h in the absence or presence of 20 *μ*M of apigenin, and then cell morphology was observed using inverted microscope. After exposure to cisplatin, HK-2 cells were damaged and significant reduction of cell density was observed (Figure 2(a)). On the other hand, treatment with apigenin reduced cisplatin-induced cellular damage, with no significant change in cell density. In apigenin-treated cells, cellular damage was not observed, but reduction of cell density was observed. To compare the percentage of apoptotic cells (Sub-G1 peak) in HK-2 cells treated with cisplatin in the absence or presence of apigenin, flow cytometric analysis was performed. The percentage of apoptotic cells measured after cisplatin treatment for 24 h was 22.5%, while it was significantly reduced to 12.7% in the presence of apigenin (Figure 2(b)). Additionally, apigenin induced cell cycle arrest at S and G2/M phases. The percentage of S and G2/M phases in apigenin-treated cells was increased from 8.7% to 22.3% and 16.6% to 22.3%, respectively, in comparison to nontreated cells. These results indicate that apigenin inhibits not only cisplatin-induced cytotoxicity in HK-2 cells but also cell proliferation.

### 3.2. Apigenin Reduces Cisplatin-Induced Caspase-3 Activity and PARP Cleavage in HK-2 Cells

**Figure 3:**
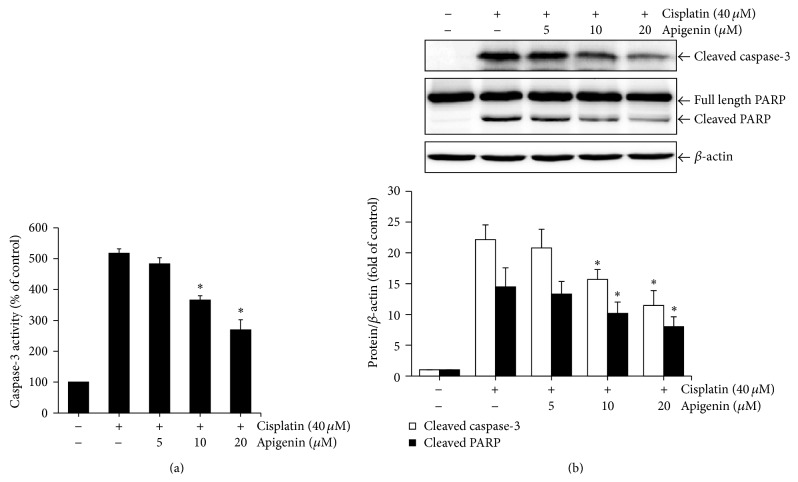
The effects of apigenin on cisplatin-induced caspase-3 activation and PARP cleavage in HK-2 cells. Cells were pretreated with different concentrations of apigenin for 1 h and then exposed to 40 *μ*M of cisplatin for 24 h. Caspase-3 activity was measured using a caspase-3 colorimetric assay kit. Values are means ± SD, *N* = 3. ^*∗*^ *p* < 0.05 versus cells treated with cisplatin alone (a). The levels of cleaved caspase-3 and PARP were examined by Western blot analysis using anticleaved caspase-3 (p20) and anti-PARP antibodies. The density of band was quantified by a densitometry using Bio-Rad Quantity One software. Value are means ± SD, *N* = 3. ^*∗*^ *p* < 0.05 versus cells treated with cisplatin alone (b).

To further determine the cytoprotective effects of apigenin against cisplatin-induced apoptotic cell death of HK-2 cells, we examined the activation of caspase-3, which plays a key role in execution of apoptosis [24] and the cleavage of poly (ADP-ribose) polymerase (PARP), which is a well-known substrate of activated caspase-3 [25]. The cells were incubated with 40 *μ*M of cisplatin for 24 h after pretreatment with different concentrations of apigenin, and then caspase-3 activation and PARP cleavage were determined by using caspase-3 colorimetric assay kit and Western blot analysis, respectively. As shown in Figure 3, treatment with apigenin significantly reduced the activity of caspase-3 and the levels of cleaved caspase-3, which is activated form of caspase-3, in cells exposed to cisplatin. In cells treated with 20 *μ*M apigenin plus cisplatin, caspase-3 activity was reduced by almost half as compared to that in cisplatin-treated cells (Figure 3(a)). Similarly, apigenin reduced proteolytic cleavage of PARP, leading to a concentration-dependent decrease in accumulation of its cleaved form (Figure 3(b)). These results indicate that apigenin may be protective against apoptotic cell death induced by cisplatin in HK-2 cells.

### 3.3. Apigenin Reduces Cisplatin-Induced Phosphorylation and Expression of p53 in HK-2 Cells

**Figure 4:**
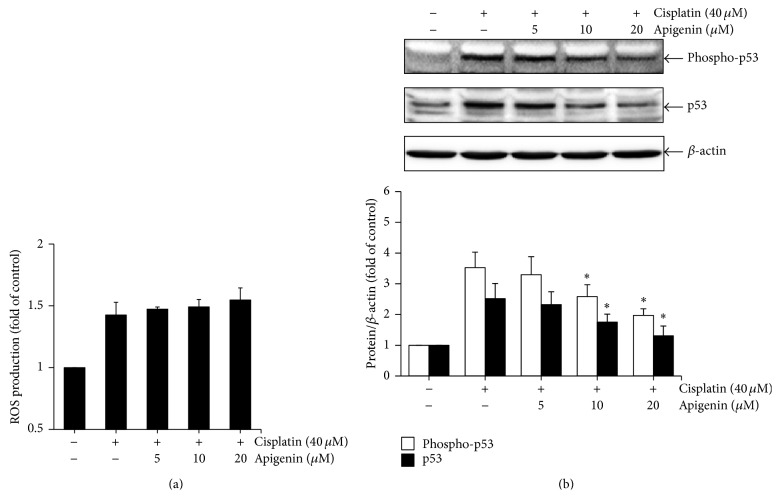
The effects of apigenin on cisplatin-induced ROS production and p53 activation in HK-2 cells. The cells were pretreated with different concentrations of apigenin for 1 h and then exposed to 40 *μ*M of cisplatin for 4 h (ROS production assay) or 8 h (Western blot analysis). The Intracellular ROS production was measured using DCFH-DA fluorescence dye. Values are means ± SD, *N* = 3 (a). The levels of p53 phosphorylation and expression were examined by Western blot analysis using anti-phospho-p53 (Ser15) and anti-p53 antibodies. The density of band was quantified by a densitometry using Bio-Rad Quantity One software. Value are means ± SD, *N* = 3. ^*∗*^ *p* < 0.05 versus cells treated with cisplatin alone (b).

Cisplatin-induced apoptosis in renal cells is associated with ROS-mediated p53 activation [9, 10]. To determine the role of ROS production and p53 activation in the cytoprotective effects of apigenin, we examined the effects of apigenin on ROS production and p53 activation after exposure to cisplatin in HK-2 cells. The cells were incubated with 40 *μ*M of cisplatin for 8 h after pretreated with different concentrations of apigenin, and then intracellular ROS production and phosphorylation level of p53 were determined by measuring the fluorescence intensity of 2′,7′-dichlorofluorescin (DCF) and Western blot analysis, respectively. The levels of ROS production were not significantly different between cisplatin-treated cells and apigenin plus cisplatin-treated cells (Figure 4(a)). However, the levels of phosphorylated p53, which is directly associated with p53 activation, were significantly reduced in cells exposed to cisplatin in the presence of apigenin (Figure 4(b)). These results indicate that apigenin may suppress cisplatin-induced apoptotic cell death in HK-2 cells through the inactivation of p53 with no significant change in ROS production.

### 3.4. Apigenin Ameliorates Cisplatin-Induced Apoptosis by Promoting Phosphorylation of Akt in HK-2 Cells

**Figure 5:**
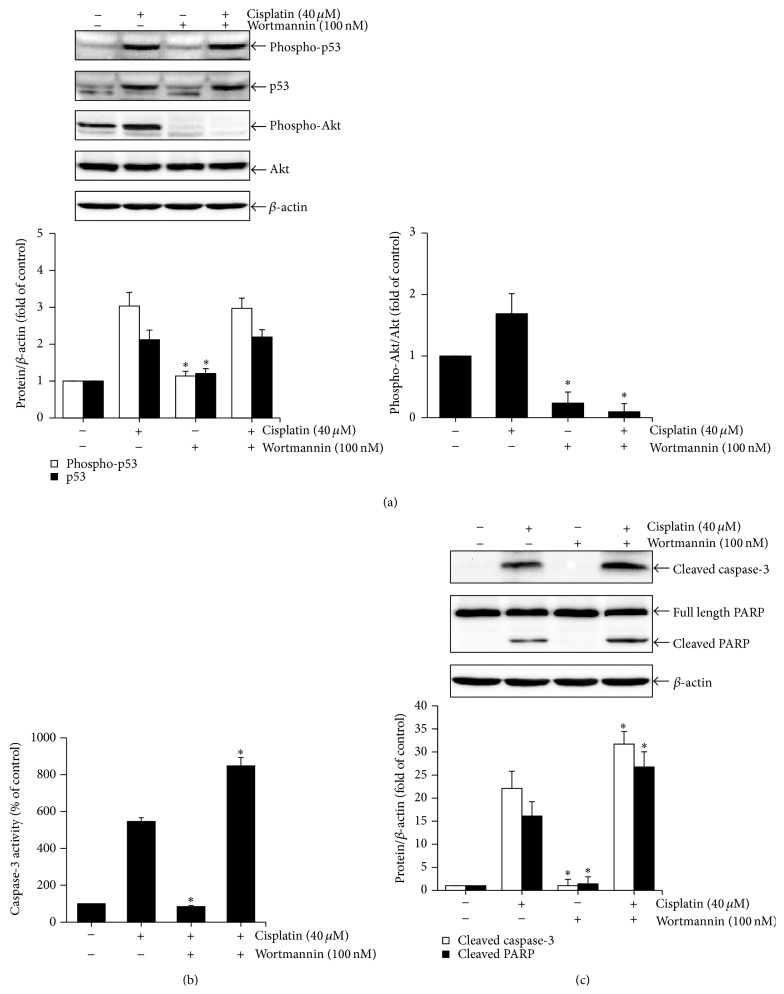
The effect of wortmannin on cisplatin-induced apoptosis in HK-2 cells. Cells were pretreated with or without 100 nM of wortmannin for 1 h and then exposed to 40 *μ*M of cisplatin for 8 h (a) or 24 h (b and c). The phosphorylation levels of p53 and Akt were examined by Western blot analysis using anti-phospho-p53 (Ser15) and anti-phospho-Akt (Ser473) antibodies. Expression of total p53 and Akt was also identified using anti-p53 and anti-Akt antibodies (a). Caspase-3 activity was measured using a caspase-3 colorimetric assay kit. Values are means ± SD, *N* = 3. ^*∗*^ *p* < 0.05 versus cells treated with cisplatin alone (b). The levels of cleaved caspase-3 and PARP were examined by Western blot analysis using anticleaved caspase-3 (p20) and anti-PARP antibodies (c). The density of band was quantified by a densitometry using Bio-Rad Quantity One software. Value are means ± SD, *N* = 3. ^*∗*^ *p* < 0.05 versus cells treated with cisplatin alone.

**Figure 6:**
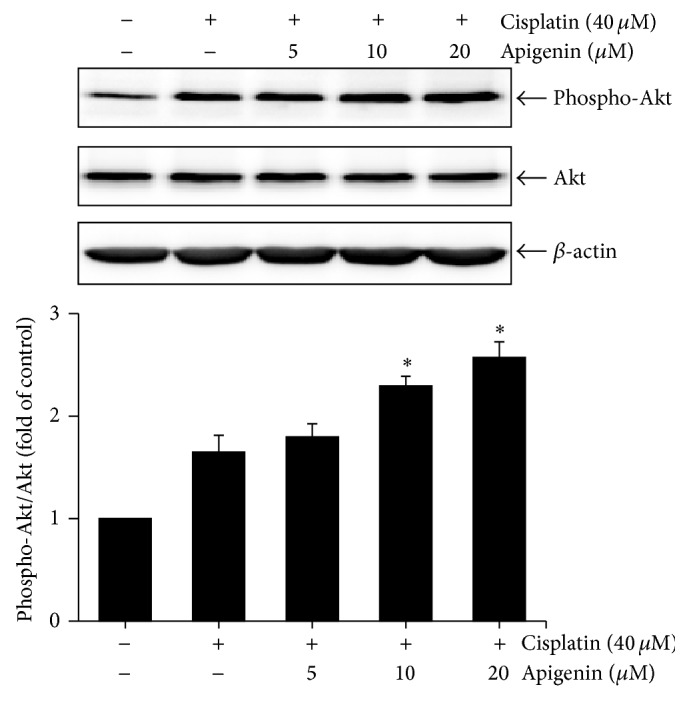
The effect of apigenin on cisplatin-induced Akt phosphorylation in HK-2 cells. Cells were pretreated with different concentrations of apigenin for 1 h and then exposed to 40 *μ*M of cisplatin for 8 h. The levels of Akt phosphorylation and expression were examined by Western blot analysis using anti-phospho-Akt (Ser473) and anti-Akt antibodies. The density of band was quantified by a densitometry using Bio-Rad Quantity One software. Value are means ± SD, *N* = 3. ^*∗*^ *p* < 0.05 versus cells treated with cisplatin alone.

The phosphatidylinositol-3 kinase (PI3K)/Akt pathway is a well-known critical signaling pathway that regulates multiple cellular processes, including cell proliferation, growth, survival, and motility [26–28]. The PI3K/Akt pathway promotes ubiquitination and degradation of p53 protein [29]. Cisplatin time-dependently increased the levels of phosphorylated Akt, indicating activation of the PI3K/Akt pathway, in HK-2 cells (data not shown). However, it was unclear whether the PI3K/Akt pathway would influence cisplatin-induced p53 activation in HK-2 cells. To clarify the role of PI3K/Akt pathway on cisplatin-induced p53 activation in HK-2 cells, we examined the effect of wortmannin, a specific inhibitor of PI3K, on phosphorylation and expression of p53 in response to cisplatin. The cells were treated with cisplatin in the absence or presence of wortmannin, and then phosphorylation levels of Akt were determined by Western blot analysis. As shown in Figure 5(a), wortmannin completely blocked Akt phosphorylation, with no significant change in phosphorylation and expression of p53, indicating that p53 activation was not associated with the PI3K/Akt pathway. We next examined the effects of wortmannin on caspase-3 activation and PARP cleavage by cisplatin treatment. Treatment with wortmannin significantly reduced the activity of caspase-3 and the cleaved forms of caspase-3 and PARP in cells exposed to cisplatin (Figures 5(b) and 5(c)). These results indicate that PI3K/Akt pathway may protect HK-2 cells, regardless of p53 activation, against cisplatin-induced apoptotic cell death. On the basis of these results, we finally examined the effect of apigenin on Akt phosphorylation after exposure to cisplatin in HK-2 cells. The cells were incubated with 40 *μ*M of cisplatin for 8 h after pretreatment with different concentrations of apigenin, and then phosphorylation levels of Akt was determined by Western blot analysis. As shown in Figure 6, treatment of apigenin strongly increased the level of phosphorylated Akt in cells exposed to cisplatin. The ratio of phospho-Akt/total-Akt in cells treated with 20 *μ*M apigenin and cisplatin was 1.6-fold higher than that in cells treated with cisplatin alone. These results indicate that apigenin may enhance cytoprotective ability of HK-2 cells against cisplatin cytotoxicity by promoting PI3K/Akt pathway.

## 4. Discussion

Nephrotoxicity is one of the main side effects of the anticancer drug cisplatin and also one of its main therapeutic limitations [1–6]. Apigenin is a low-toxic dietary flavonoid abundantly found in fruits and vegetables and has antioxidant and anticancer activities [11–21]. In the present study, we demonstrated for the first time the protective effect of apigenin on human renal proximal epithelial cells against cisplatin-induced apoptosis. Our results show that apigenin inhibited cisplatin-induced apoptosis in HK-2 cells. Interestingly, apigenin itself inhibited cell growth because of its ability to induce cell cycle arrest at S and G2/M phases. In line with this, apigenin has been previously reported to inhibit cell proliferation and induces cell cycle arrest in various cell types [30–33].

Cisplatin induces apoptotic cell death in renal cells through ROS-mediating p53 activation [9, 10]. ROS scavengers, such as NAC, and p53 inhibitors, such as pifithrin-*α*, inhibit cisplatin-induced apoptosis by the inactivation of p53 in renal cells. Even if apigenin is known to have antioxidant activity [14], apigenin, as shown in our results, inhibited cisplatin-induced p53 phosphorylation and expression, with no significant reduction of ROS production in HK-2 cells. Thus, it is most likely that apigenin may inhibit cisplatin-induced apoptosis in HK-2 cells by inactivating p53 but not by reducing ROS production.

Akt (or protein kinase B) is a well-characterized serine/threonine kinase that is the central protein in the PI3K/Akt pathway and promotes cellular survival [34]. Akt enhances Mdm2-mediated ubiquitination and degradation of p53 protein [29]. The oncoprotein Mdm2 is a ubiquitin ligase E3 for p53 protein and allows export p53 from the nucleus to the cytoplasm where it targets p53 for degradation [35, 36]. Our results show that cisplatin induced p53 activation in HK-2 cells, but at the same time it induced Akt activation. We thought that Akt activation would partially inhibit cisplatin-induced p53 activation in HK-2 cells. However, the inactivation of Akt by PI3K inhibitor had no effect on p53 activation induced by cisplatin in HK-2 cells. Instead, we found that inhibition of Akt activation promoted cisplatin-induced apoptotic cell death in HK-2 cells. Previously reported studies have shown that Akt promotes cisplatin resistance through inhibition of p53 phosphorylation in cancer cells [37, 38]. On the other hand, Skladanowski et al. reported that inhibition of Akt in p53-deficient cells promotes apoptotic cell death induced by cisplatin [39]. Zhang et al. also reported that Akt knockdown increases cell susceptibility to cisplatin, which is p53-independent [40]. Thus, it is most likely that PI3K/Akt pathway may protect HK-2 cells from undergoing apoptosis induced by cisplatin cytotoxicity or contribute to cisplatin resistance. In our study, apigenin promoted cisplatin-induced Akt phosphorylation in HK-2 cells. In other words, the enhanced activation of Akt by apigenin seems to contribute to protect HK-2 cells against cisplatin cytotoxicity. Taken together, these results suggest that apigenin may be beneficial as anticancer supplement in alleviating cisplatin-induced nephrotoxicity.

## 5. Conclusion

Apigenin ameliorates cisplatin-induced nephrotoxicity in HK-2 cells through reduction of p53 activation and promotion of PI3K/Akt pathway but inhibits cell growth because of its ability to induce cell cycle arrest at S and G2/M phases. In addition, apigenin suppresses cisplatin-induced p53 activation and apoptosis, which is not associated with its antioxidant activity because of no effect on ROS production. However, it is unclear how apigenin can inhibit cisplatin-induced p53 activation. Further studies are required to identify possible mechanisms by which apigenin can inhibit p53 activation induced by cisplatin in HK-2 cells.
